# Novel *IL2RG* Gene Mutation in One of Dizygotic Twins Causing Profound Changes of Receptor Structure

**DOI:** 10.3389/fped.2022.858166

**Published:** 2022-04-15

**Authors:** Magdalena Rutkowska-Zapała, Anna Szaflarska, Anna Kluczewska, Julia Ciȩciwa, Jacek Plewka, Anna Michalska, Maciej Siedlar

**Affiliations:** ^1^Department of Clinical Immunology, Institute of Paediatrics, Jagiellonian University Medical College, Kraków, Poland; ^2^Department of Clinical Immunology, University Children Hospital, Krakow, Poland; ^3^Malopolska Centre of Biotechnology, Jagiellonian University, Kraków, Poland

**Keywords:** X-linked severe combined immunodeficiency, interleukin 2 receptor gamma, dizygotic twins, novel genetic variant, protein structure

## Abstract

In this study, we report a 4-month-old boy with T^−^B^+^NK^−^ severe combined immunodeficiency (SCID) due to a novel mutation in exon 2 of *IL2RG*, the gene encoding the interleukin (IL) common gamma chain (γc) of the cytokine receptors for IL-2, IL-4, IL-7, IL-9, IL-15, and IL-21. The patient was born from a twin pregnancy. He manifested recurrent infections of the gastrointestinal tract, whereas his twin brother was asymptomatic with no immune defects. In order to evaluate the effect of this unreported variant on the protein structure, a structural modeling process was performed showing prominent biochemical alterations of the protein features, including molecular weight, isoelectric charge, and possible changes to its secondary and tertiary structure.

## Introduction

Severe combined immunodeficiency (SCID) is a group of inherited disorders responsible for life-threatening dysfunctions of the immune system. Among them, inherited in an X-linked manner SCID (SCIDX-1, X-SCID) is the most prevalent, accounting for approximately half of the cases of SCID, with an estimated incidence of 1:130,000 live births (1). SCIDX-1 is caused by mutations of *IL2RG*, the gene encoding a common gamma chain (also known as γc or CD132), which is a transmembrane protein shared by cytokine receptors for interleukin (IL)-2, IL-4, IL-7, IL-9, IL-15, and IL-21 (2, 3). The *IL2RG* gene spans 4.5 kb of genomic DNA organized in 8 exons and encodes a protein of 369 amino acids. The γc molecule is expressed on the cell surface of lymphoid, myeloid cells, and hematopoietic progenitors (4, 5). It consists of three domains: extracellular (carries the gene for family conserved cysteines and repeated tryptophan and serine motifs), transmembrane, and intracellular. Together with the IL-2Ra and IL-2Rb subunits, γc composes the cellular receptor for IL-2 and is essential for its signal transduction through activation of JAK3 tyrosine kinase (6, 7). So far, according to the VarSome database, 284 known variants in the *IL2RG* gene are observed, of which 147 are pathogenic, 51 are uncertain, and 86 are benign (8). The most common pathogenic variants identified in affected patients are missense mutations located mainly in the extracellular domain encoded by exons 1 to 5 of *IL2RG*, followed by nonsense and frameshift mutations (1, 7, 9).

In this study, we report a 4-month-old boy with T^−^B^+^NK^−^ SCID due to an unreported nonsense mutation in exon 2 of the *IL2RG* gene. The patient was derived from a twin pregnancy, and his twin brother was asymptomatic with no immune defects. In order to confirm the pathogenic effect of the detected novel variant on the protein structure, a modeling process was performed.

## Materials and Methods

### Patient

Our patient was the third child of non-consanguineous parents. The second-born male twin, after 37 weeks of pregnancy, was delivered by cesarean section, weighing 2,520 g. The Apgar score was 10 at 1 and 5 min. The pregnancy was uneventful, and there was no relevant family history, specifically no history of primary immunodeficiencies in both families. He was vaccinated against bacille Calmette–Guérin (BCG) and hepatitis B in the first 24 h of life. The infant was asymptomatic until 1 month of life when he was admitted to the hospital because of diarrhea with dehydration. No pathogens in stools were detected, but the C-reactive protein (CRP) level was increased leading to the antibiotic treatment (amoxicillin with clavulonic acid). The second hospitalization took place only a few days after the first, once again because of diarrhea with no detectable pathogens in stools and with an increased CRP level. He again received an antibiotic (cefuroxime). Because a heart murmur was detected on the physical examination and in ECHO atrial septal defect (ASD) II, the patient was given enalapril. Moreover, he had bilateral free inguinal hernias. When he was 2 months old, he again developed diarrhea and was admitted to the Children's University Hospital of Cracow. The patient received azithromycin therapy as the campylobacter antigen was detected in his stool samples. The next day, he developed a severe norovirus infection with dehydration, hypoalbuminemia, and metabolic disturbances. Additionally, the patient was diagnosed with an *Escherichia coli* urinary tract infection that was treated with cefuroxime. Despite no visible respiratory issues, the patient's chest X-ray showed mild interstitial changes. Viral infections, including human immunodeficiency virus (HIV), cytomegalovirus (CMV), and Epstein–Barr virus (EBV), were excluded with the PCR method. We tested three gastric lavage samples, which were negative for acid-fast bacilli (AFB) and for Mycobacterium tuberculosis (DNA probe and culture). During his stay, he was consulted by a cardiologist, an endocrinologist, and a surgeon. Because of the prolonged diarrhea, he received additional co-trimoxazol treatment. He was fed with protein hydrolysates (Neocate). At that time point, the patient was referred to our immunology ward where additional immunological tests were performed. The parameters analyzed showed decreased levels of immunoglobulin G (IgG) and immunoglobulin A (IgA), a normal level of immunoglobulin M (IgM), and a lack of T lymphocytes and natural killer (NK) cells, whereas the number of B lymphocytes was within normal ranges. The chest X-ray and ultrasound examination did not reveal thymus presence. We also performed flow cytometric analysis in the patient‘s twin brother, who showed a normal absolute number of T, B, and NK cells (Tables 1, 2). We also proved dizygosity of the two brothers performing twin chimerism analysis. Our patient was admitted without diarrhea, showed no symptoms of an infection, and had no pathogens detected in his blood and urine samples. However, norovirus antigens were present in the stool samples. Antibiotics, piperacillin/tazobactam, cefuroxime, and also co-trimoxazole, micafungin, rifampicin with isoniazid as BCG-itis prophylaxis, and IVIG substitution (every 10 days in a 500 mg/kg dose) were included in the therapeutic schedule. After excluding DiGeorge syndrome, on the basis of clinical and laboratory findings, X-linked SCID was the putative diagnosis. Therefore, the *IL2RG* gene was selected for Sanger sequencing analysis. The patient is currently being prepared for hematopoietic stem cell transplantation (HSCT) from his human leukocyte antigen (HLA)-matched twin brother.

**Table 1:** The number of white blood cells (WBCs) and their population at first examination in the patient and his twin brother.

	**Patient**		**Patient's twin brother**		**Age-matched** **reference values**	
WBCs (cells/μl x 10^−3^)	9.69		7.18		5.98 - 13.51	
	**% of WBCs**	**Cells/μl x 10** ^**−3**^	**% of WBCs**	**Cells/μl x 10** ^**−3**^	**% of WBCs**	**Cells/μl x 10** ^**−3**^
Lymphocytes	22.1	2.14	66.4	4.77	26.0–85.6	1.52–8.99
Neutrophils	17.1	1.66	18.9	1.35	10.9–76.0	0.97–7.21
Eosinophils	46.9	4.54	3.3	0.24	0.0–4.0	0.02–0.82
Monocytes	13.3	1.29	10.7	0.77	3.8–13.4	0.24–1.17

**Table 2:** Humoral and cellular immunity parameters at first examination in the patient and his twin brother.

**Parameter**	**Patient**		**Patient's twin brother**		**Age-matched reference values**	
**Serum immunoglobulins (g/l)**
IgG	1.56		nd		2.66 - 7.01	
IgA	<0.07		nd		0.07 - 0.37	
IgM	0.45		nd		0.17 - 0.86	
**Lymphocyte subpopulations**	**% of lymphocytes**	**Cells/μl**	**% of lymphocytes**	**Cells/μl**	**% of lymphocytes**	**Cells/μl**
CD3	0.1	2	64	3052	53.0–84.0	2500–5500
CD4	0.1	2	52	2479	35.0–64,0	1600–4000
CD8	0	0	9	429	12–28	560–1700
CD3/HLADR	0.1	2	2.1	100	5.0–8.0	135–232
CD19	98	2098	29	1383	6–32	300–2000
CD3-16+56+	1	21	6	286	4–18	170–1100

*nd, not determined*.

### Immunological Assays

All immunological parameters were determined during routine laboratory tests. The concentration of the serum immunoglobulin level was measured by nephelometry, whereas the percentage and absolute numbers of a particular subpopulation of lymphocytes were determined by flow cytometry.

### Molecular Analysis

#### DNA Isolation

Genomic DNA was isolated using a QIAmp DNA Mini Kit (Qiagen, Hilden, Germany) from peripheral blood drawn into tubes containing ethylenediaminetetraacetic acid (EDTA) as an anticoagulant. DNA concentrations and quality were measured using a Quawell Q5000 UV–vis spectrophotometer.

#### Sanger Sequencing of the *IL2RG* Gene

Coding sequences of the *IL2RG* gene were amplified by PCR, which was performed using 100 ng of spectrophotometrically quantified DNA, 0.3 units of AmpliTaq Gold polymerase (Thermo Fisher Scientific), 0.5 mM of each deoxynucleoside triphosphate (dNTP), 2.5 mM MgCl_2_, and 0.5 l M of each primer (Genomed, Poland) specific to all 8 exons of the *IL2RG* gene. The reaction volume was 20 μl. Primer sequences for amplification and sequencing reactions are available on request. The PCR products were purified with an ExoSAP-IT For PCR Product Cleanup (Affymetrix) according to the manufacturer's instructions. Then, Sanger sequencing PCR was performed using the BigDye Terminator v3.1 Cycle Sequencing Kit (Thermo Fisher Scientific) and exon-specific primer. PCR products were purified by ethanol/EDTA precipitation and dissolved in Hi-Di Formamide (Thermo Fisher Scientific), denatured at 95°C for 3 min, and run on the Applied Biosystems 3500 Genetic Analyzer with the use of POP-7 polymer, 50 cm capillary, default run parameters, and analyzed with the Sequencing Analysis software v5.4 (Applied Biosystems). For each exon, bidirectional sequence analysis was performed. The DNA sequencing results were aligned to the *IL2RG* gene sequence available in the Ensembl database (reference sequences ENSG00000147168; the numbering of exons and mutation nomenclature were according to the transcript ENST00000374202.7).

#### Chimerism Analysis

The presence of chimerism among the twin brothers was assessed using short tandem repeat (STR) genotyping and an AmpFLSTR^TM^ Identifiler^TM^ PCR Amplification Kit (Thermo Fisher Scientific). The kit amplifies 16 loci including amelogenin in a single tube and provides loci corresponding to the STR database standards. Fluorescently labeled PCR products were capillary electrophoresed on the sequencing apparatus ABI Prism 3,500 with the use of POP-7 polymer and 50 cm capillary. The resulting signals were analyzed with Peak Scanner^TM^ Software v1.0.

### Structural Analysis

To elucidate the molecular interactions of the mutated protein, a high-resolution model was generated using the Muster software. The model was later compared with the available wild-type model of *IL2RG* made using AlphaFold (PDB ID: A1xS) (10).

## Results

**Figure 1:**
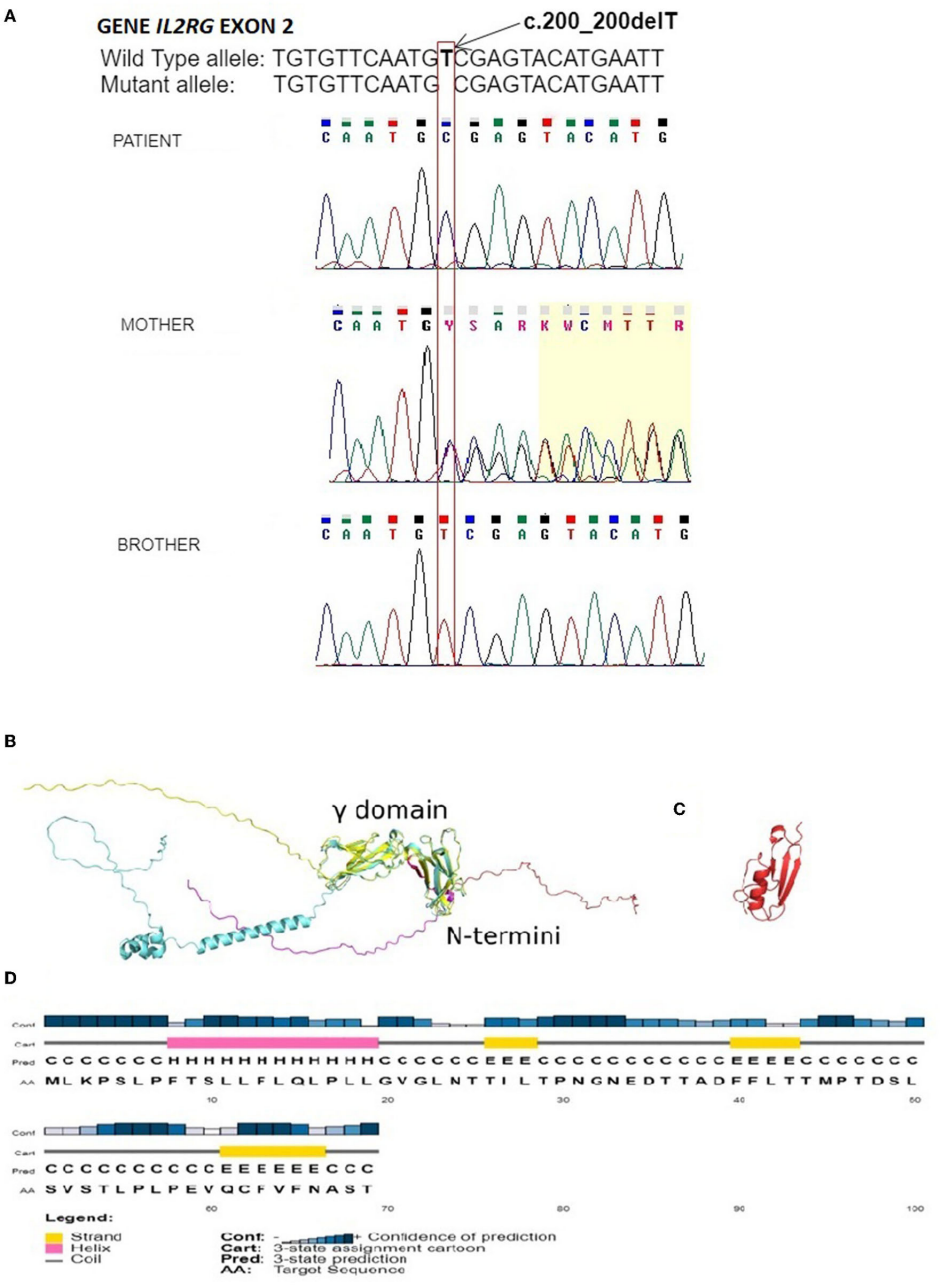
**(A)** Electropherogram of the fragment of the *IL2RG* gene's second exon of the patient, showing unreported deletion c.200_200delT, resulting in frameshift variant V67Afs*4. Electropherogram of the patient's mother confirms the carrier of the nucleotide variant observed in our patient. The correct sequence of the analyzed fragment of the gene in the healthy twin brother is shown at the bottom. Positions of the mentioned nucleotide are indicated by the frame with an arrow. The sequence fragments were displayed by the Chromas Lite software; **(B)** AlphaFold (cyan) and Muster (yellow) models of the *IL2RG* with the mutant variant sequence in red and magenta; **(C)** The Muster model reported a mutant variant of *IL2RG* showing secondary structures; and **(D)** PsiPred prediction of secondary structures with their probabilities based on the mutant variant sequence of *IL2RG*.

In the genetic material of our patient, a novel unreported hemizygous deletion of T nucleotide in exon 2 of this gene was detected. This variant, localized at position c.200_200delT (cDNA.292_292delT; g.1143_1143delT), leads to a frameshift followed by a premature stop codon (p.V67Afs^*^4). In order to determine the inheritance of the detected variant, the sequence analysis of exon 2 of the *IL2RG* gene in the patient's mother was performed, and her heterozygous state was confirmed. Since the twin brother was selected as the bone marrow donor, the sequence analysis of exon 2 of the *IL2RG* gene was also performed on it, and the results revealed no pathogenic variant in the analyzed region. Results of the patient's and his family's DNA sequencing are shown in Figure 1A.

In the next step, we generated a model of the high-resolution structure of IL2RG protein based on the mutated sequence found in our patient and compared it to the available model from AlphaFold (PDB ID: A1xS) (11). The comparison revealed that the structure of the γ domain within *IL2RG* that was reported to be involved in a complex formation with IL-2Rα and IL-2Rβ (PDB ID: 2ERJ) was conserved in both models, whereas both N and C termini were intrinsically disordered. The product of partial expression of IL2RG reported here comprised the first 69 amino acids that were within the disordered N terminus (Figure 1B, red color). The analysis of the secondary structures predicted for this region (Figure 1C) suggested the presence of twelve amino acid long α-helices and three short β-sheets, which were also present in the model of mutated IL2RG; however, the level of the prediction confidence was low (Figure 1D). Biochemically, such truncation of IL2RG not only significantly reduces its molecular weight from 42 to 7.5 kDa but also alters its isoelectric point from slightly acidic pI 5.9 to 3.8, severely modifying its charge distribution.

## Discussion

In this study, we described a patient with clinical features of SCID, in whom a novel unreported variant in the *IL2RG* gene (c.200_200delT) was detected. It is worth mentioning that within the codon where the patient's variant was localized, two other gene variants have been described: substitution c.201C>T (rs756100347) and substitution c.199G>A (rs775243259), with benign and uncertain significance status, respectively. Interestingly, our patient was derived from a twin pregnancy, and his twin brother was asymptomatic with no immune defect. According to our best knowledge, this is the first report concerning X-SCID in one of dizygotic twins.

Based on the clinical picture, it can be assumed that the novel mutation observed in our patient is pathogenic. Moreover, it is possible that the clinical picture of our patient is concerned with this particular type of mutation. Infections in this child were mainly related to the gastrointestinal tract. However, we performed only gastric lavages and cannot definitely rule out BCG infection in the intestines. Among 20 SCID children diagnosed in our center from 2000, 5 of them have had T^−^B^+^NK^−^ concerned with *IL2RG* gene mutation. All these cases revealed both pulmonary and gastrointestinal tract infections. Some of these patients demonstrated BCG-itis, whereas, in the described twin, no symptoms were observed.

The modeling process also seems to confirm the pathogenicity of the detected variant. Structurally, the truncated variant of *IL2RG* does not have a γ domain responsible for the interactions with IL-2Rα and IL-2Rβ, therefore preventing the assembly of IL-2 receptor ectodomains. Moreover, the resulting mutant, even though it is most likely disordered, has the potential to have a tertiary structure. This, in combination with a significantly more negatively charged surface, may allow the mutant variant to interact with other proteins resulting in the further alteration of the immune system.

In conclusion, the novel genetic variant detected in our patient may cause structural changes in receptor protein, which result in the phenotype of T^−^B^+^NK^−^ X-linked SCID.

## Data Availability Statement

The datasets for this article are not publicly available due to concerns regarding participant/patient anonymity. Requests to access the datasets should be directed to the corresponding author.

## Ethics Statement

Ethical review and approval was not required for the study on human participants in accordance with the local legislation and institutional requirements. Written informed consent to participate in this study was provided by the participants' legal guardian/next of kin.

## Author Contributions

MR-Z and AS were responsible for the patient's clinical care, planned the diagnostic procedures, and wrote the manuscript. AK and JC performed the sequencing analysis and contributed to the final version of the manuscript. JP performed and described the structural analysis of the mutated protein. AM performed chimerism analysis. MS supervised the study and data analysis and revised the article. All authors contributed to the article and approved the submitted version.

## Funding

This work was supported by the O Zdrowie Dziecka Foundation of the University Children's Hospital in Cracow.

## Conflict of Interest

The authors declare that the research was conducted in the absence of any commercial or financial relationships that could be construed as a potential conflict of interest.

## Publisher's Note

All claims expressed in this article are solely those of the authors and do not necessarily represent those of their affiliated organizations, or those of the publisher, the editors and the reviewers. Any product that may be evaluated in this article, or claim that may be made by its manufacturer, is not guaranteed or endorsed by the publisher.
